# ESP32-S3-Based Multimodal Wearable for Remote Vital-Sign Acquisition with ECG-to-PPG Pulse-Arrival-Time Tracking and Functional Cloud Triage: An Engineering Proof of Concept

**DOI:** 10.3390/s26196047

**Published:** 2026-09-24

**Authors:** Mohamed Ali Torad, Hamdy Elsayed Ahmed, Ahmed Ali Mohamed Torad, Mona Mohamed Taha, András Attila Horváth, Mohamed Abdelaziz Emam

**Affiliations:** 1 Department of Electronics and Communication Engineering, Higher Technological Institute (HTI), 10th of Ramadan City 44634, Egypt; 20210909@hti.edu.eg; 2 Department of Physical Therapy and Health Rehabilitation, Jouf University, Sakaka 72341, Saudi Arabia; almtorad@ju.edu.sa; 3 Department of Basic Sciences, Faculty of Physical Therapy, Kafrelsheikh University, Kafrelsheikh 33516, Egypt; 4 Department of Rehabilitation Sciences, College of Health and Rehabilitation Sciences, Princess Nourah Bint Abdulrahman University, P.O. Box 84428, Riyadh 11671, Saudi Arabia; 5 Neurocognitive Research Centre, Nyírő Gyula National Institute of Psychiatry and Addictology, 1135 Budapest, Hungary; 6 Department of Anatomy, Histology and Embryology, Semmelweis University, 1085 Budapest, Hungary; 7 János Szentágothai Neurosciences Division, Semmelweis University, 1085 Budapest, Hungary

**Keywords:** wearable sensors, ESP32-S3, electrocardiography, photoplethysmography, pulse-arrival time, remote patient monitoring, Internet of Medical Things, cloud triage

## Abstract

**Highlights:**

**What are the main findings?**

- A single ESP32-S3 supported synchronized ECG, PPG/SpO2, and contact-temperature acquisition while FreeRTOS isolated physiological sampling from network activity.
- System-level engineering verification demonstrated synchronized multimodal acquisition, functional propagation of tested synthetic critical scenarios, sub-second dashboard presentation in development logs, and automatic recovery from temporary Wi-Fi interruption. No human-participant data contributed to the results reported in this proof-of-concept manuscript.

**What are the implications of the main findings?**

- The work demonstrates an end-to-end low-cost remote-monitoring proof of concept spanning wearable sensing, dual-core firmware, cloud storage, role-based dashboards, and human-in-the-loop triage.
- Clinical deployment requires prospective human-participant validation with reference-grade instrumentation, formal cuffless blood-pressure calibration and agreement analysis, reproducible classifier reporting, quantitative timing/reliability and power testing, and broader safety and cybersecurity evaluation.

**Abstract:**

Continuous remote physiological monitoring requires low-cost sensing nodes to preserve timing fidelity while maintaining secure connectivity and interpretable escalation. This study presents an engineering proof-of-concept platform centered on an ESP32-S3 with AD8232 ECG, MAX30102 PPG/SpO2, and MAX30205 contact-temperature sensing. FreeRTOS separates nominal 100 Hz ECG/PPG acquisition from network communication, and the ECG R-wave-to-peripheral-PPG-foot interval is treated as pulse-arrival time (PAT). Telemetry is transmitted over authenticated HTTPS to a Laravel platform, while a separate FastAPI service provides human-in-the-loop normal/warning/critical functional triage. The current evidence is limited to bench-top engineering operation and synthetically constructed triage scenarios; no human-participant measurements are analyzed or reported. An ethics-approved rest-exertion-recovery protocol has been prepared for a future validation stage. The present results support system integration and engineering feasibility, while physiological accuracy, PAT-to-blood-pressure calibration, respiratory-rate agreement, classifier performance, timing reliability, and battery/power characteristics remain to be established quantitatively.

## 1. Introduction

Remote patient monitoring has evolved from isolated sensor demonstrations into end-to-end Internet of Medical Things (IoMT) architectures that combine physiological acquisition, wireless communication, storage, visualization, and increasingly automated analytics [1,2]. This transition is especially relevant when frequent bedside observation is burdensome, when patients are geographically distant from specialist care, or when minimizing unnecessary staff–patient contact is desirable. Low-cost physiological monitoring can also expand access in resource-constrained settings, provided that affordability does not substitute explicit validation and safety boundaries.

A central engineering challenge is preserving physiological timing while a low-cost wearable simultaneously performs wireless communication and cloud exchange. This is important when synchronized ECG and PPG waveforms are used to derive pulse-timing features. The original firmware referred to the interval from the ECG R peak to the peripheral PPG pulse foot as pulse transit time (PTT). An ECG-to-peripheral-pulse interval strictly includes the cardiac pre-ejection period and is more accurately described as pulse-arrival time (PAT) [3,4,5]. This manuscript therefore uses PAT for the implemented timing feature.

In this manuscript, “low-cost” is used qualitatively to describe the deliberate selection of widely available commodity components—ESP32-S3, AD8232, MAX30102, MAX30205, an 18650 battery subsystem, indicators/buzzer, and prototyping-board hardware. No priced bill of materials, measured total prototype cost, or quantitative cost-effectiveness advantage is claimed.

Source-document cost boundary: The graduation-project source report contains broader economic language, including “small fraction” comparisons with hospital-based monitoring. Those source-report statements are not carried forward as quantitative findings here because the retained submission record does not contain a priced bill of materials, a measured total prototype cost, or an auditable cost-effectiveness comparison. In this manuscript, “low-cost” therefore remains a qualitative descriptor of commodity-component selection only.

The present work evaluates whether a single ESP32-S3 can support synchronized multimodal sensor acquisition while maintaining authenticated cloud communication, role-specific web presentation, and a functional cloud-triage pipeline. The architecture combines an AD8232 ECG front end, MAX30102 PPG/SpO2 sensing, MAX30205 contact-temperature sensing, a FreeRTOS dual-core firmware layer, a Laravel web platform, and a separately deployed FastAPI triage service. The study is intentionally framed as an engineering proof of concept rather than a clinically validated medical device, and no human-participant data are used in Section 4.

The contribution is primarily at the systems-integration and architecture level rather than a new physiological sensing principle or a newly validated machine-learning algorithm. Specifically, the work demonstrates: (i) consolidation of sensor acquisition and network-gateway functions on one dual-core ESP32-S3; (ii) shared-clock ECG/PPG acquisition for beat-level PAT tracking; (iii) a web-first, role-differentiated cloud platform for clinicians, relatives, and administrators; and (iv) a decoupled, human-in-the-loop cloud-triage microservice with graceful fallback behavior. The current evaluation is restricted to engineering integration, synthetic alert propagation, dashboard presentation, and qualitative endurance observations.

## 2. Literature Review

### 2.1. IoT Healthcare Monitoring Architectures

IoT healthcare systems are commonly organized as layered architectures that connect a sensing or perception layer to network transport and application-level storage, analytics, and visualization [1]. Cloud integration extends this model by separating data acquisition from analysis and allowing computational services to evolve independently of the embedded sensor node [2]. For wearable monitoring, this separation is attractive because the resource-constrained device can prioritize deterministic acquisition while higher-level analytics, user management, and historical data services remain in the cloud.

Photoplethysmography is a well-established optical method for tracking pulsatile blood-volume changes and supports heart-rate and oxygen-saturation sensing in wearable devices [6]. When synchronized with ECG, PPG can also supply a peripheral pulse timing landmark for pulse-arrival analysis. However, the cuffless blood-pressure literature emphasizes that ECG-referenced PAT is not identical to vascular PTT and that any blood-pressure use requires careful calibration and validation [3,4,5].

### 2.2. Closely Related Wearable and IoMT Systems

Heaney et al. developed an IoT ECG and vital-sign monitoring system using an AD8232 front end, temperature sensing, MAX-series pulse oximetry, local/mobile visualization, MATLAB connectivity, and a web interface; the authors reported 99.56% accuracy relative to their commercial reference device [7]. Georgieva-Tsaneva et al. combined synchronized ECG and PPG in an IoT cardio-monitoring platform for heart-rate-variability and arrhythmia-related assessment, including time- and frequency-domain cardiac analysis; cuffless blood pressure was not the target application [8].

Contardi et al. demonstrated direct integration of a MAX30102 photometric biosensor with ESP32 web-server capabilities for continuous heart-rate and oxygen-saturation monitoring, supporting the feasibility of using an ESP32 as both acquisition and connectivity hardware without a separate gateway [9]. Adochiei et al. focused on interoperability, using ESP32-based acquisition with HL7 FHIR-oriented data representation and reporting sub-second end-to-end flow with zero packet loss across ten acquisition sessions [10].

Torad et al. presented an isolation-ward monitoring prototype that used Arduino-based acquisition, Raspberry Pi edge processing, Firebase cloud storage, LabVIEW/Flutter interfaces, and a 1D-CNN ECG classifier trained on the MIT-BIH Arrhythmia Database; the study reported sub-200 ms inference and 300–800 ms round-trip cloud latency [11]. The prior system is an architectural precedent for the remote-monitoring motivation, but its implementation differs materially from the present single-ESP32-S3/Laravel/FastAPI architecture.

Direct comparison with the authors’ earlier Sensors prototype [11] clarifies the distinction. Hardware: The earlier system split acquisition and processing across Arduino and Raspberry Pi hardware, whereas the present design combines acquisition and network-gateway functions on one ESP32-S3. Software: The earlier stack used Firebase, LabVIEW, and Flutter, whereas the present stack uses authenticated HTTP/JSON, Laravel browser interfaces, and a separately deployable FastAPI service. Algorithms/data: The earlier work used 1D-CNN ECG arrhythmia classification with the MIT-BIH dataset; the present manuscript uses PAT tracking and a ten-feature cloud-triage artifact whose retained training documentation is incomplete, so only functional pipeline behavior is claimed. Experiments: The current manuscript reports bench-top engineering and synthetic-scenario results and contains no human-participant dataset. The retained project documentation identifies conceptual and architectural influence from the earlier work but does not provide a code-overlap audit; code-level reuse is therefore not quantified or claimed.

### 2.3. Research Gap and Positioning of the Present System

The reviewed systems establish feasibility for low-cost physiological sensing, ESP32-based web connectivity, standards-oriented IoMT transport, and automated analysis. The present study does not claim a new sensor principle or a new clinically validated classifier. Its specific contribution is the integration, within one architecture, of shared-clock ECG/PPG acquisition for PAT tracking, a single-board ESP32-S3 acquisition-and-gateway design, role-differentiated browser-based monitoring, and a separately deployable cloud-triage boundary. This is a targeted feature and architecture comparison rather than a systematic review, and the novelty claim is therefore limited to the implemented systems-integration choices summarized in Table 1.

The technical contribution is therefore not any individual sensor, microcontroller, or classifier in isolation, but the coordinated systems-engineering implementation of: (i) shared-clock ECG/PPG acquisition for PAT tracking; (ii) dual-core temporal isolation of physiological sampling from network activity; (iii) single-board acquisition-and-gateway consolidation; and (iv) role-based web workflows coupled to a separately deployable triage service with graceful fallback behavior.

## 3. Methodology

### 3.1. System Architecture and Design Requirements

The system was designed as a layered sensing-to-cloud pipeline (Figure 1). The wearable node acquires ECG, PPG/SpO2, and contact-temperature signals; the ESP32-S3 performs time-critical acquisition, local status management, and Wi-Fi communication; a Laravel backend receives authenticated telemetry and persists it to a relational database; and a separately deployed FastAPI service returns a normal, warning, or critical triage label. Browser-based interfaces expose different information to clinicians, relatives, and administrators. The principal design requirements were continuous sensor acquisition, isolation of network activity from timing-critical sampling, secure telemetry transport, low hardware cost, and graceful degradation if the analytic service or network connection became unavailable.

### 3.2. Wearable Hardware and Sensor Interfaces

The prototype integrates an AD8232 single-lead ECG analog front end [12], a MAX30102 PPG/SpO2 module [13], and a MAX30205 digital contact-temperature sensor [14] around an ESP32-S3 dual-core microcontroller with integrated Wi-Fi [15]. The AD8232 analog output is connected to GPIO 4, while LO+ and LO− leads-off signals are monitored on GPIO 5 and GPIO 18. The MAX30102 and MAX30205 share an I2C bus on GPIO 8 (SDA) and GPIO 9 (SCL). A rechargeable 18650-based power subsystem supplies the device, and firmware-controlled RGB indicators and a buzzer provide local status and alert feedback. The implemented hardware channels and ESP32-S3 interfaces are summarized in Table 2, and the assembled proof-of-concept hardware is shown in Figure 2.

The source project documentation occasionally describes the MAX30205 output as core body temperature. Because the present proof of concept uses superficial/contact placement and has no reference-grade core-temperature comparison, the manuscript conservatively describes this channel as contact skin/body-surface temperature. This wording describes the implemented sensing interface without implying core-temperature accuracy.

The current hardware is a perforated-board prototype powered by an 18650 lithium-ion subsystem and housed in a prototype enclosure. The retained engineering records do not provide a validated battery-capacity specification for the assembled unit, instrumented current/power measurements, continuous runtime after a full charge, or final enclosure dimensions and weight. The intended sensing arrangement uses torso ECG electrodes, a fingertip MAX30102, and contact placement of the MAX30205. These unquantified power and wearability characteristics are reported as limitations rather than inferred specifications.

### 3.3. Physiological Signal Acquisition and Processing

#### 3.3.1. ECG/PPG Shared Timing and PAT Detection

ECG and PPG acquisition are performed within the same high-priority sensor task at a nominal 100 Hz rate. This shared acquisition loop places the electrical and optical channels in a common clock domain and reduces inter-device clock drift. At 100 Hz, the raw sample spacing is 10 ms. The firmware record does not document sub-sample interpolation or a measured sampling-jitter distribution; consequently, the present PAT value has 10 ms sample-level quantization and is treated as an engineering-resolution timing feature.

Implementation-record cross-check shows that ECG and PPG are acquired in the same TaskSensors loop on a shared clock; R-peak and PPG-foot timestamps use the same monotonic hardware timer; and development logging confirmed that the nominal 100 Hz sampling cadence was maintained with Wi-Fi active. These observations support the synchronization design but do not replace a measured jitter distribution, missed-sample rate, or repeatability analysis.

For ECG landmark detection, the retained implementation record describes a lightweight Pan-Tompkins-like sequence: a derivative operation emphasizes the QRS slope, the result is squared, a short moving-window integrator smooths the response, and a rolling adaptive amplitude threshold identifies candidate R peaks. Candidate peaks with implausibly high amplitude relative to the recent rolling level are rejected as likely motion artifact. The corresponding peripheral PPG pulse foot is then identified in the synchronized optical waveform, and PAT is computed once per accepted cardiac cycle from the R-peak sample to the PPG-foot sample using the same time base. The retained record does not preserve a numerical PPG-foot criterion or an annotated validation trace, which limits independent reproduction of the exact landmark detector.

The 10 ms sampling step is not assumed to be negligible relative to physiologically relevant beat-to-beat PAT changes. A near-term firmware improvement is therefore to quantify timing jitter and repeatability and to implement validated sub-sample landmark refinement (for example, local parabolic/spline interpolation or an explicitly defined derivative-based PPG-foot rule) before PAT is used for reference-based blood-pressure calibration.

#### 3.3.2. PAT-Based Blood-Pressure Trend Model for Future Calibration

The software architecture defines two separate empirical PAT-to-pressure trend relationships so that the systolic and diastolic fields supplied to the cloud-triage vector are not derived from a single indistinguishable scalar output. The same PAT input is used with independently fitted coefficient pairs for systolic and diastolic trend estimates. These coefficients are intended to be fitted in a future participant-specific calibration study using paired PAT/reference-cuff observations; no participant coefficient pair is estimated or reported in the current manuscript.

Source-document clarification: The graduation-project report describes preliminary per-subject PTT/reference-cuff coefficient fitting during laboratory trials. The associated participant-level paired measurements and fitted coefficients are not reported in the present submission as an auditable dataset and are not used to generate or validate any result in this manuscript. Accordingly, the equations below define only the planned PAT calibration interface for a future prospectively documented study; no source-report subject-specific calibration is claimed as a validated result here.

(1) $${SBP}_{trend}=a_{sys}+b_{sys}\ln PAT/1\;\mathrm{ms}$$

(2) $${DBP}_{trend}=a_{dia}+b_{dia}\ln PAT/1\;\mathrm{ms}$$

In Equations (1) and (2), PAT is the ECG-to-PPG interval and PAT/1 ms is a dimensionless numerical ratio. When the models are fitted against cuff pressure in mmHg, $a_{sys}$, $b_{sys}$, $a_{dia}$, and $b_{dia}$ carry mmHg so that the resulting trend fields are expressed in mmHg. The future calibration protocol must define the number of paired cuff observations, estimate the two coefficient pairs separately, and reserve independent data for performance assessment (or use a pre-specified cross-validation design). Until those data exist, the equations define the planned calibration interface only and do not constitute validated blood-pressure estimation.

#### 3.3.3. Respiratory-Rate Inference from ECG and PPG

Respiratory rate is inferred from the synchronized ECG and PPG channels rather than from a dedicated respiratory sensor. Respiration modulates ECG-derived features such as R-R interval and beat morphology and modulates the PPG baseline, pulse amplitude, and pulse-to-pulse interval [16,17]. The retained implementation record describes electrode-contact, pulse-signal-strength, and valid-beat quality checks; processing within a sliding analysis window; isolation of a low-frequency band appropriate for resting adult breathing; candidate-cycle detection; and quality-weighted fusion of ECG-derived respiration (EDR) and PPG-derived respiration (PDR). When one channel is degraded by motion artifact or dropout, the valid channel is prioritized and confidence is reduced. However, the exact analysis-window duration, numerical band limits, and fusion weights are not preserved in the retained development documentation; these parameters must be fixed and reported in a reproducible future implementation. Because no respiratory-belt, capnography, or other reference comparison is reported, the current output is described only as an unvalidated respiratory-rate trend.

### 3.4. FreeRTOS Dual-Core Firmware

The ESP32-S3 firmware uses FreeRTOS [18] to isolate network activity from sensor acquisition (Figure 3). Core 1 hosts TaskSensors at priority 5 and TaskUI at priority 3, while Core 0 hosts TaskNetwork at priority 2. A bounded inter-task queue transfers prepared ECG/PPG samples, PAT-related timing data, and timestamps from TaskSensors to TaskNetwork; the sensor task uses a non-blocking queue handoff so a full queue cannot stall the 100 Hz acquisition loop, while the network task drains and batches data independently. The firmware also implements watchdog recovery, diagnostic logging, hysteresis for status indicators, and automatic Wi-Fi reconnection.

This task and core separation is intended to reduce timing interference from variable-duration Wi-Fi, DNS, HTTP, or cloud operations. It provides architectural temporal isolation, but it is not itself a quantitative jitter guarantee; sampling jitter, missed-sample rate, queue-overflow rate, and timing repeatability have not yet been characterized with instrumented measurements.

### 3.5. Cloud Platform, Database, and Implemented Access-Control Safeguards

Telemetry is transmitted from the device as structured JSON over authenticated HTTPS to a Laravel web application [19]. The backend persists incoming readings in a relational database and associates them with patient, clinician, relative/family, and device records. Role-based middleware restricts access to the appropriate dashboard and workflow. Parameterized database queries, CSRF protection for state-changing web requests, and one-way password hashing are implemented as application-level safeguards. These measures describe the implemented security controls; the study does not claim formal cybersecurity validation, penetration testing, or medical-device security certification.

The clinician interface exposes a patient roster, trend data, alerts, clinical notes, and communication functions; the relative interface intentionally simplifies telemetry into status-oriented gauges and trends; and the administrator interface manages users, devices, and platform-level operations. The architecture keeps acquisition, persistence, presentation, and analytic inference as separately maintainable layers.

### 3.6. Cloud-Triage Microservice

A Python V.3.12.10/FastAPI V.0.139.0 microservice provides a pluggable cloud-triage layer. The deployed implementation loads a static scikit-learn/joblib model artifact (real_final_health_model.pkl) that was trained offline on labeled ten-feature vectors. Retained records describe a training/held-out-test split, feature normalization, comparison of candidate classifier families, and deployment of a serialized artifact, but they do not preserve the dataset source, exact sample count, class distribution, final estimator family, hyperparameters, or sufficient held-out metrics for independent reproduction. The component is therefore evaluated only as a functional inference-and-alert pipeline. Accordingly, independent reproduction of the classifier is not claimed.

For avoidance of doubt, the serialized classifier is treated in this manuscript as a legacy functional component of the pipeline rather than as an independently reproducible or clinically validated predictive model.

Based on the triage input appears in Table 3, the triage output prioritizes human review rather than making an autonomous diagnosis or treatment decision. The development implementation uses an 80% severe-class probability threshold for a critical label; this threshold is a heuristic configuration value and was not selected through ROC analysis, calibration analysis, or a clinical sensitivity study. If the FastAPI service is unavailable, Laravel continues to store data and can fallback to basic threshold alerting, preserving raw physiological visibility.

Implementation-record cross-check note: retained project documentation gives conflicting descriptions of the two blood-pressure trend inputs—one describes a planned future PAT calibration interface, while another describes subject-specific PTT/reference-cuff fitting. Pending reconciliation of the underlying implementation and ethics records, these two classifier fields are treated here only as planned PAT-derived trend inputs, and no participant-calibrated blood-pressure output is claimed.

The source report’s statement that coefficients were fitted from subject-specific cuff observations is therefore acknowledged as a preliminary project record, but it is not used to establish the provenance, calibration validity, or clinical accuracy of the two BP trend fields in this manuscript.

The ten-feature input also creates unresolved model-audit questions. In particular, hypertension status, diabetes status, smoking status, age, and gender may influence the output independently of live sensor features; no retained feature-importance analysis, ablation study, or age/gender performance-disparity analysis is available. The manuscript therefore does not attribute a synthetic critical alert to PAT, blood-pressure trend fields, or any specific sensor feature. Future model work must document dataset provenance, class balance, estimator and hyperparameters, confusion matrices and class-specific metrics, probability calibration, feature ablation/importance, and subgroup/fairness analyses before predictive-performance claims are made.

### 3.7. Software Presentation Layer

Figure 4 combines two software-layer views. Figure 4a is a conceptual consolidation of modules and data flows that were implemented across the platform; its layout is illustrative rather than a literal deployment schematic. Figure 4b shows the implemented 24 h report interface generated by the cloud-triage workflow. These views document software architecture and decision-support presentation.

Figure 5 shows a representative clinician patient-detail screen with live vital-sign cards and waveform trends. The higher-level Doctor Dashboard and Alerts page are retained as the principal triage views (Figure 6 and Figure 7).

On the clinician side, the Doctor Dashboard organizes assigned demonstration records as risk-coded cards and surfaces recent high-priority events for rapid review (Figure 6), while the Alerts page provides severity-based filtering and acknowledgement (Figure 7). The subscription-management page has been moved to Supplementary Figure S7. All names, identifiers, physiological values, and alert labels visible in the main and supplementary interface figures are synthetic demonstration records used solely for software and pipeline testing; they are not human-participant data. Supplementary Figure S7 corresponds to the Subscription and Billing Control screen in the source project document.

### 3.8. Planned Human-Participant Validation Protocol (Future Work)

Study No. KFSIRB200-1196 defines a planned future human-participant validation procedure consisting of approximately five minutes of seated rest, a brief supervised period of light step-up exercise, and approximately five minutes of seated recovery. The intended instrumentation is non-invasive AD8232 ECG electrodes(Analog Devices, Inc. Wilmington, MA, USA), a MAX30102 fingertip PPG/heart-rate/SpO2 sensor(Analog Devices, Inc. Wilmington, MA, USA), a MAX30205 contact-temperature sensor(Analog Devices, Inc. Wilmington, MA, USA), and identified reference instruments including a validated blood-pressure cuff. Synchronized ECG and PPG would be retained for PAT analysis. This protocol is described to define the next validation stage; no participant session or participant-derived measurement contributes to the results reported in this manuscript.

The Scientific Research Ethics Committee of Kafrelsheikh University documented final approval of Study No. KFSIRB200-1196 on 27 August 2026. Consent materials aligned with the approved procedure were prepared for four prospective volunteers. Written informed consent is required before any future participant measurements are collected or used. Because the current manuscript contains no human-participant results, there are no participant measurement dates, participant characteristics, or participant-level reference-device comparisons to report for the present study.

Relationship to the source graduation report: The graduation-project source document describes preliminary healthy-volunteer laboratory activities, including HR/SpO2 comparison with commercial wearables, rest-exertion observations, and PTT/reference-cuff calibration activities. Those descriptions are treated here as historical project-development records, not as participant evidence for the present paper. The retained materials available for this submission do not provide a complete auditable participant-level record—such as raw paired measurements, trial dates, participant characteristics, reference-device identifiers, paired PAT/cuff observations, and fitted calibration coefficients—sufficient for journal-grade analysis. Consequently, no volunteer-derived measurement from the source report is analyzed, tabulated, or used to support Section 4; the approved protocol described above defines the future human-participant validation stage for this manuscript.

The future validation study should predefine participant number and eligibility, reference-device manufacturer/model, measurement schedule, repeated paired observations, calibration and independent assessment sets, and the statistical analysis plan. Heart rate, SpO2, and contact temperature should be compared observation-by-observation with reference devices; PAT-based systolic/diastolic trend models should be calibrated against reference cuff measurements; respiratory-rate inference should be compared with a respiratory belt, capnography, or equivalent reference; and all participant-derived results must be collected after ethics approval and written consent.

### 3.9. Triage, Alert-Latency, and Endurance Tests

The triage path was exercised using normal-range engineering inputs and synthetically constructed out-of-range feature vectors designed to cross one or more configured risk thresholds. The objective was to verify functional classifier/alert-path behavior—classification, persistence, and alert propagation—rather than machine-learning sensitivity, specificity, or clinical prediction. The exact injection point used for every historical synthetic vector is not preserved, so these tests are not described as full sensing-path end-to-end tests. The number and composition of the historical synthetic scenario set were not retained as a reproducible test dataset. The 80% severe-class confidence threshold is therefore treated as a heuristic development setting, not a validated clinical cutoff.

Separately, end-to-end device-to-dashboard alert presentation was documented for a critical-threshold reading generated on the ESP32-S3 and propagated through the Laravel API, FastAPI inference, persistence, and dashboard presentation. Development logs summarize this path as sub-second but do not provide a raw latency distribution, number of repeated trials, or mean/maximum latency. Multi-hour engineering operation and deliberately induced Wi-Fi outages were also observed qualitatively, with no recorded task starvation or watchdog reset and automatic reconnection without manual restart. Packet loss, missed samples, queue overflows, reconnection-time distributions, power consumption, and battery runtime were not measured quantitatively and are therefore not reported as performance benchmarks.

### 3.10. Statistical Analysis, Reporting Boundary, and GenAI Disclosure

Reporting boundary: The current manuscript reports an engineering proof of concept only. No human-participant or clinical measurement is analyzed in Section 4; the approved participant protocol in Section 3.8 is future work. The retained records support bench-top sensor/firmware integration, synthetic triage-path testing, and qualitative endurance observations, but do not contain paired reference-instrument data for physiological error statistics, a reproducible classifier evaluation dataset, or quantitative jitter/reliability/power measurements. Consequently, the manuscript makes no claim of physiological accuracy, cuffless blood-pressure accuracy, respiratory-rate accuracy, classifier sensitivity/specificity, subgroup fairness, or regulatory-grade device performance. Manufacturer specifications are distinguished explicitly from measured system results.

## 4. Results and Discussion

### 4.1. Integrated System Operation and Principal Findings

The completed prototype operated as an integrated acquisition-to-dashboard engineering pipeline during bench-top development. Sensor streams were sampled on the ESP32-S3 while network communication proceeded on the opposite core, and the cloud platform stored incoming measurements, invoked functional triage, and exposed results to role-specific interfaces. Local system and battery indicators remained available independently of the web interface, and the staged integration strategy allowed the sensor/firmware, Laravel, and FastAPI subsystems to be tested independently before full-pipeline testing.

This section contains no human-participant observations. Engineering verification is limited to synchronized multimodal acquisition, independent network communication, synthetic high-risk alert propagation, dashboard presentation summarized in development logs as sub-second, and automatic recovery from temporary network interruption. These findings are interpreted at the system-function level defined in Section 3.10.

### 4.2. Sensor-Channel Functional Verification and Validation Status

The respiratory-rate pipeline was exercised as an integrated signal-processing function: EDR and PDR paths produced candidate trend estimates within the implemented analysis workflow, and the fusion stage selected or combined them according to signal-quality evidence. No certified respiratory reference was used in the current study, and the retained records do not support a respiratory-rate error statistic.

No quantitative reference-device agreement is reported for heart rate, SpO2, ECG waveform, or contact temperature. The MAX30205 value of ±0.1 °C is treated only as a manufacturer-rated sensor specification, not a system-level measured error. The AD8232 leads-off and signal-acquisition paths were functionally verified, but no quantitative ECG waveform error is available. Table 4 therefore records validation status rather than accuracy. Published results from other systems are not used as a substitute for direct validation of the present prototype.

### 4.3. PAT Timing Feature and Planned Blood-Pressure Calibration

The current engineering result is the synchronized calculation path for ECG-R-peak-to-PPG-foot PAT at nominal 100 Hz; no participant rest-exertion-recovery dataset is analyzed in this paper. The planned protocol described in Section 3.8 is intended to test whether within-participant PAT changes track reference cuff pressure across rest, light exertion, and recovery, but those measurements are future work.

PAT is not equivalent to vascular PTT because ECG-referenced timing includes the cardiac pre-ejection period [3,4]. In addition, the present 10 ms sample spacing, unquantified sampling jitter, and lack of validated sub-sample landmark interpolation constrain the precision of the current timing feature. These limitations must be addressed before formal PAT-to-pressure calibration is interpreted physiologically.

No PAT-versus-reference-pressure scatterplot, paired error distribution, correlation analysis, Bland–Altman analysis, or calibration-stability statistic is reported because no participant reference dataset is included. Future work should estimate separate systolic and diastolic calibration coefficients, use independent assessment data, and report bias, MAE/RMSE, limits of agreement, and stability across time, posture, activity, and participants [5].

### 4.4. Functional Cloud Triage and Alert Delivery

The deployed triage service returned class probabilities and the development workflow mapped severe-class probability above the heuristic 80% threshold to a critical status. Synthetically constructed abnormal vectors crossed the configured threshold and produced the intended dashboard alerts, demonstrating software-path behavior rather than clinical classifier performance. A documented example combined an artificially elevated heart rate with depressed SpO2.

Normal-range synthetic inputs did not produce a critical label in the retained demonstration runs. However, the number and composition of historical scenarios are not documented, so no false-positive rate is calculated. The observation is also conditional on the 80% heuristic threshold; changing that threshold could change the number of critical labels, and no threshold-sensitivity analysis is available. Because the number and full composition of the historical scenario set were not retained, these observations are not reported as sensitivity, specificity, or false-positive-rate estimates.

The ten-feature vector contains both live sensor variables and static demographic/comorbidity variables. Because no feature-importance or ablation study is available, the current evidence cannot show whether a critical output is driven by PAT-related fields or by static features such as hypertension status. Age/gender performance disparity was also not assessed. These issues are explicitly reserved for reproducible model validation and fairness analysis.

For the documented device-generated critical-threshold-reading-to-dashboard path, development logs summarize alert presentation as sub-second, but the raw trial count and latency distribution are unavailable. The synthetic-vector work is therefore reported separately as a functional classifier/alert-path observation, not as a complete sensing-path latency experiment or a journal-grade predictive-model evaluation.

### 4.5. Engineering Endurance and Interface Observations

During extended multi-hour engineering runs, no sensor-task starvation or watchdog reset was recorded, and deliberately induced temporary Wi-Fi interruptions were followed by automatic reconnection without manual restart. Because run counts, exact duration, reconnection-time distribution, packet loss, missed samples, queue overflows, and power/battery measurements were not retained quantitatively, these observations support only basic functional robustness.

The clinician and relative interfaces were designed for different information depth, with simplified status presentation for relatives and richer telemetry/alert presentation for clinicians. No formal usability or human-factors study, and no human-participant assessment of the interfaces, is reported.

### 4.6. Comparison with Related IoMT Systems

The present system shares the low-cost multi-parameter sensing direction of Heaney et al. [7] and the direct ESP32/web approach of Contardi et al. [9]. It differs from the interoperability-centered Adochiei et al. framework [10], which uses HL7 FHIR-oriented resources, and from the Torad et al. prototype [11], which distributes acquisition and edge classification across Arduino/Raspberry Pi hardware and uses Firebase/LabVIEW/Flutter interfaces. The present contribution is strongest at the systems-integration level: one dual-core ESP32-S3 provides shared-clock acquisition and connectivity, Laravel provides role-specific browser access, and FastAPI defines a separately deployable functional triage boundary.

Table 5 provides source-reported context only; protocols, populations, endpoints, and measurement conditions differ, and the values are not a controlled head-to-head benchmark. The present system row deliberately reports only the functional engineering observation supported by retained records.

### 4.7. Limitations and Future Validation

The principal limitation is that no human-participant or clinical experiment contributes data to the current results. Therefore, heart-rate/SpO2/contact-temperature agreement, ECG waveform agreement, PAT-to-blood-pressure calibration, and respiratory-rate agreement with reference instrumentation remain unestablished. The 100 Hz ECG/PPG rate produces 10 ms sample-level PAT quantization, and sampling jitter, sub-sample landmark error, missed-sample rate, and queue overflow have not been quantified. The triage classifier is not independently reproducible from the retained records, its 80% severe-class threshold is heuristic, and feature leakage/importance and age/gender disparity have not been assessed. Alert latency, packet loss, Wi-Fi reconnection time, power consumption, battery runtime, enclosure dimensions/weight, and formal wearability are also not quantitatively characterized. Finally, the prototype has not undergone electrical-safety, EMC, cybersecurity, formal usability, or medical-device regulatory validation.

The Scientific Research Ethics Committee of Kafrelsheikh University approved Study No. KFSIRB200-1196 on 27 August 2026 for the planned human-participant validation stage. Consent materials were prepared for four prospective volunteers, but no participant measurements are analyzed or reported in this manuscript. Thus, the ethics approval and consent documentation define a future experimental pathway rather than the evidentiary basis of the present engineering results.

Future work should execute a preregistered, ethics-approved validation with a predefined sample size and physiologically diverse cohort; reference-grade ECG, oximetry, temperature, respiratory, and cuff/continuous-pressure instrumentation; raw synchronized ECG/PPG retention; explicit PPG-foot and R-peak definitions; measured sampling jitter and sub-sample interpolation; paired error, bias, MAE/RMSE, confidence intervals, and agreement plots; separate calibration and assessment of systolic/diastolic PAT models; and calibration-stability analysis across posture, activity, time, and participants. The classifier dataset and training pipeline should be version-controlled and fully reproducible, with confusion matrices, class-specific metrics, calibration, ablation/feature-importance, threshold-sensitivity, and subgroup/fairness analyses. Engineering follow-up should quantify alert latency, packet loss, missed samples, queue overflows, reconnection time, current/power draw, battery runtime, device dimensions/weight, cybersecurity, and interoperability mapping to HL7 FHIR [10].

## 5. Conclusions

This study presents an integrated engineering proof-of-concept architecture for low-cost remote vital-sign acquisition using an ESP32-S3, AD8232 ECG, MAX30102 PPG/SpO2, MAX30205 contact temperature, FreeRTOS dual-core firmware, a Laravel web platform, and a separate FastAPI triage service. The retained evidence supports synchronized acquisition, functional sensor interfacing and leads-off handling, synthetic critical-alert propagation, dashboard presentation summarized as sub-second in development logs, and qualitative multi-hour reconnection behavior. No human-participant measurements are included in the reported results.

Accordingly, the contribution should be interpreted as system integration and architectural feasibility. PAT-based systolic/diastolic trend calibration, respiratory-rate accuracy, physiological sensor agreement, and predictive classifier performance require the planned reference-based human-participant study. Quantitative embedded timing, reliability, power, wearability, safety, cybersecurity, and fairness evaluations are also required before any clinical deployment claim is appropriate.

## Figures and Tables

**Figure 1:**
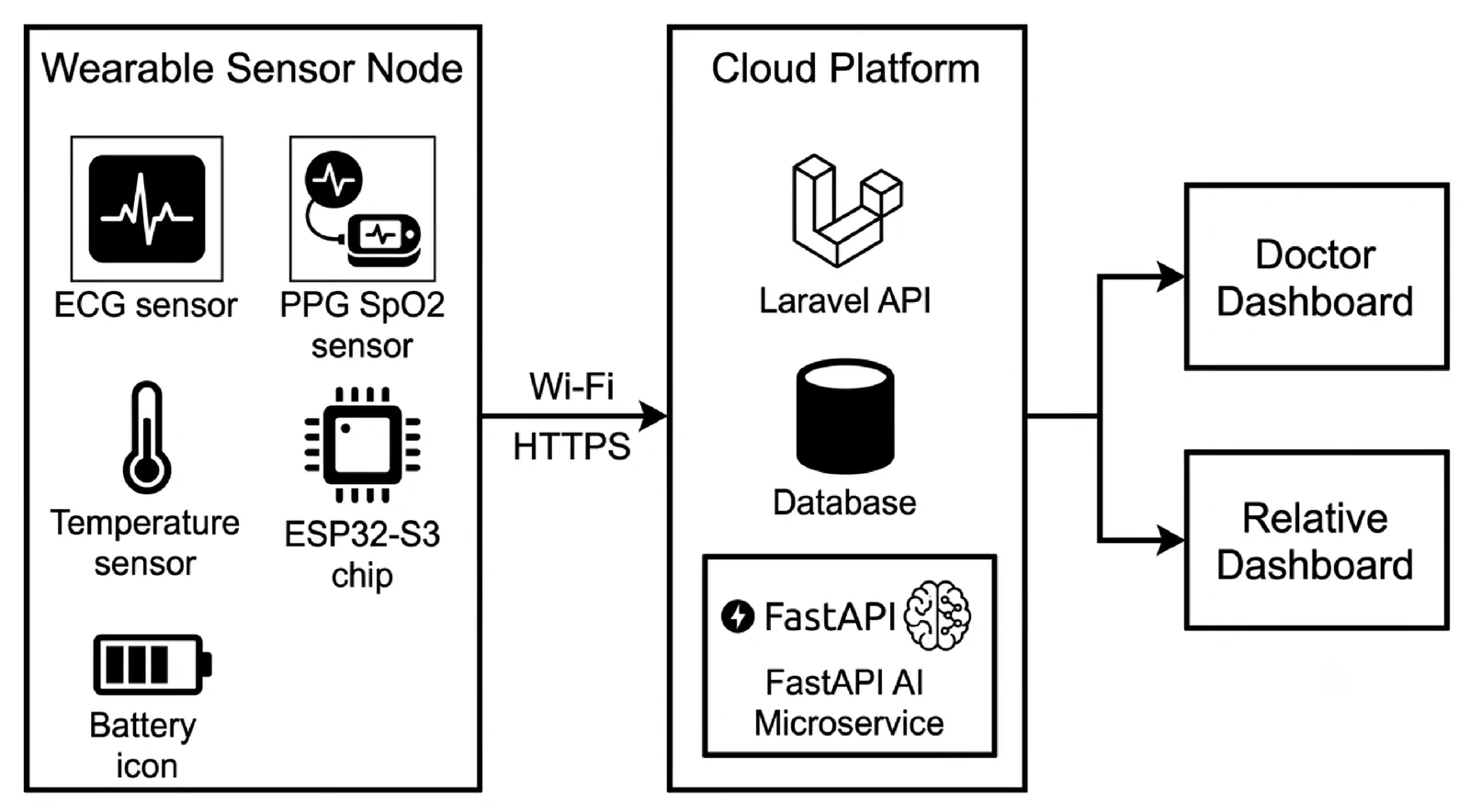
High-level architecture of the wearable sensor node, ESP32-S3 acquisition/gateway layer, cloud platform, functional triage service, and role-oriented dashboards.

**Figure 2:**
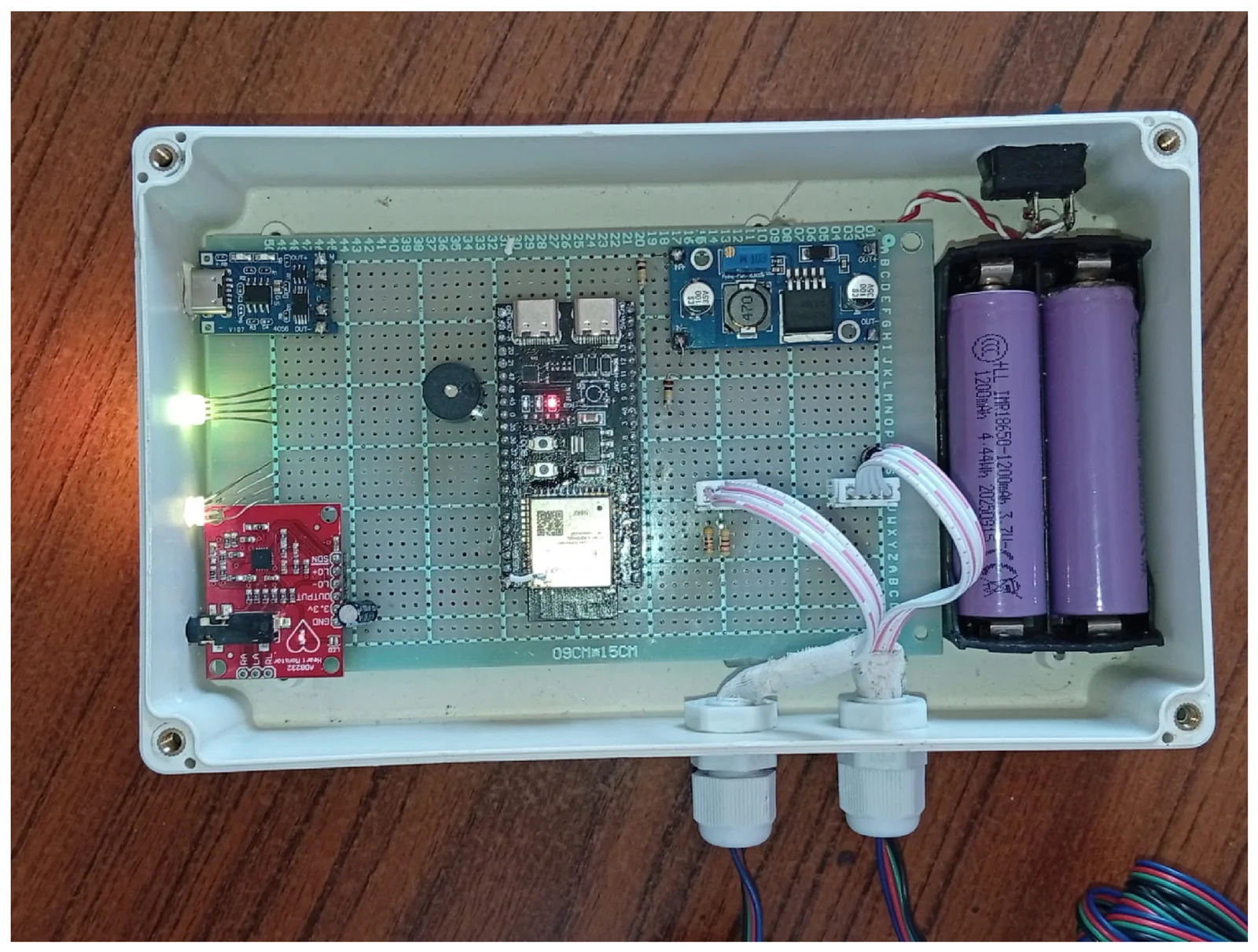
Assembled hardware proof of concept on perforated board within the prototype enclosure. The image shows the ESP32-S3, sensor-interface electronics, indicator circuitry, and battery subsystem.

**Figure 3:**
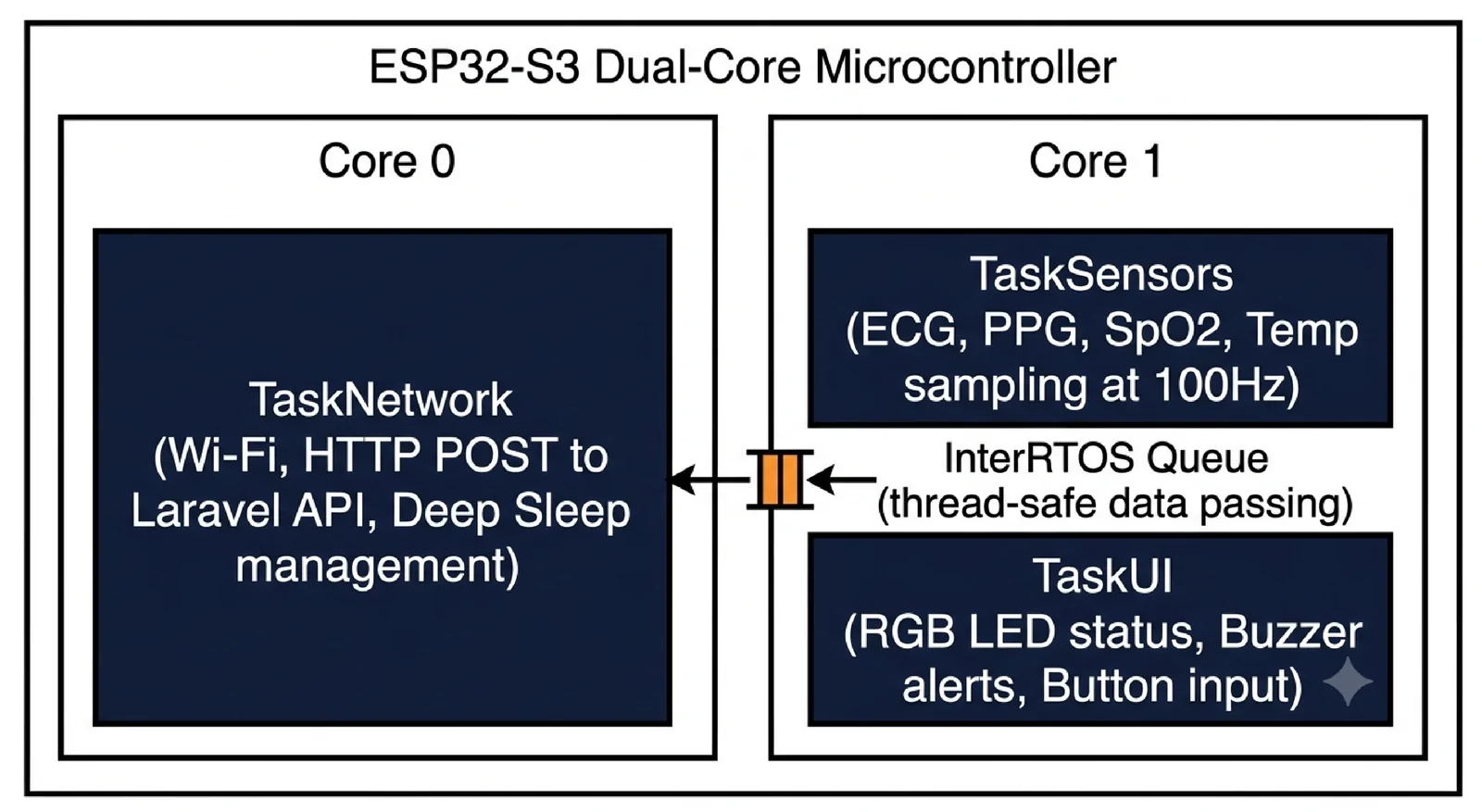
FreeRTOS dual-core task allocation used to separate time-critical physiological acquisition from network communication and user-interface activity.

**Figure 4:**
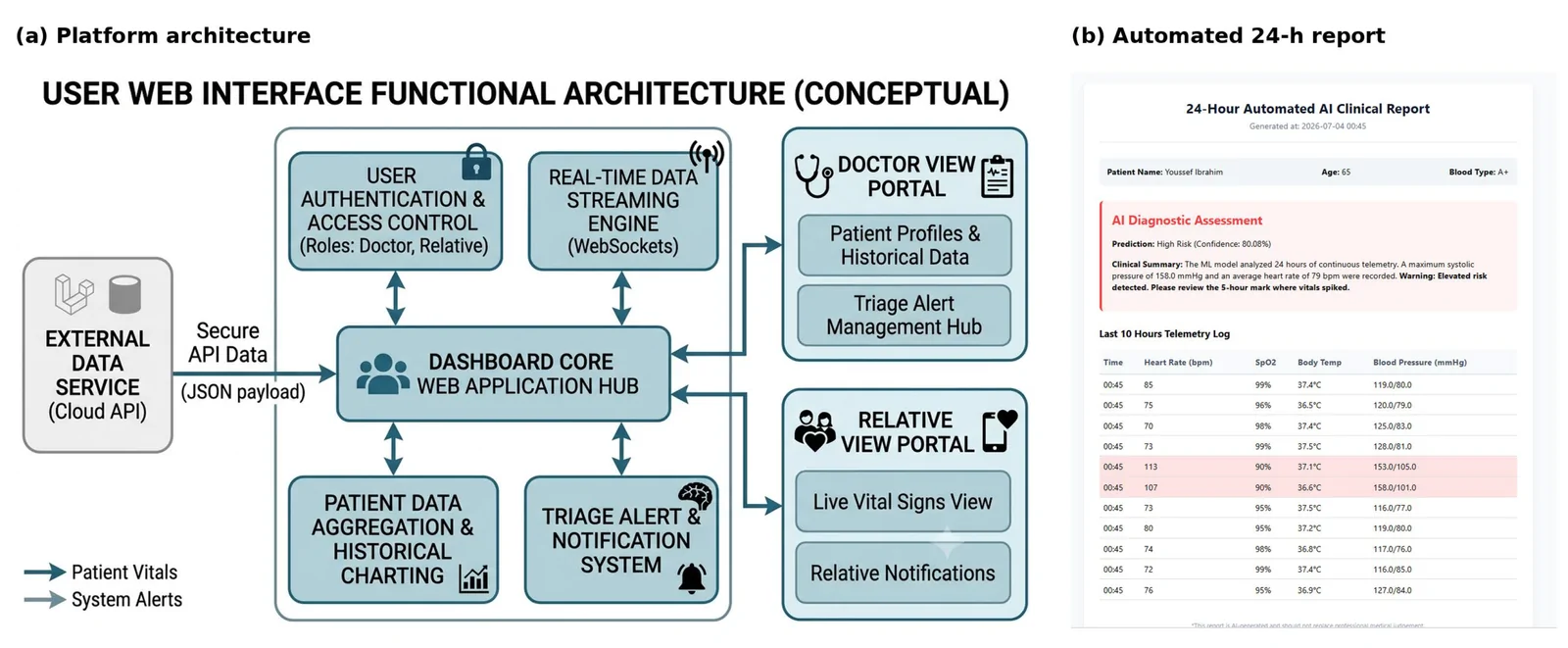
Software-layer views: (**a**) Conceptual consolidation of implemented role-oriented web modules and data flows; (**b**) example 24 h report interface generated by the cloud-triage workflow.

**Figure 5:**
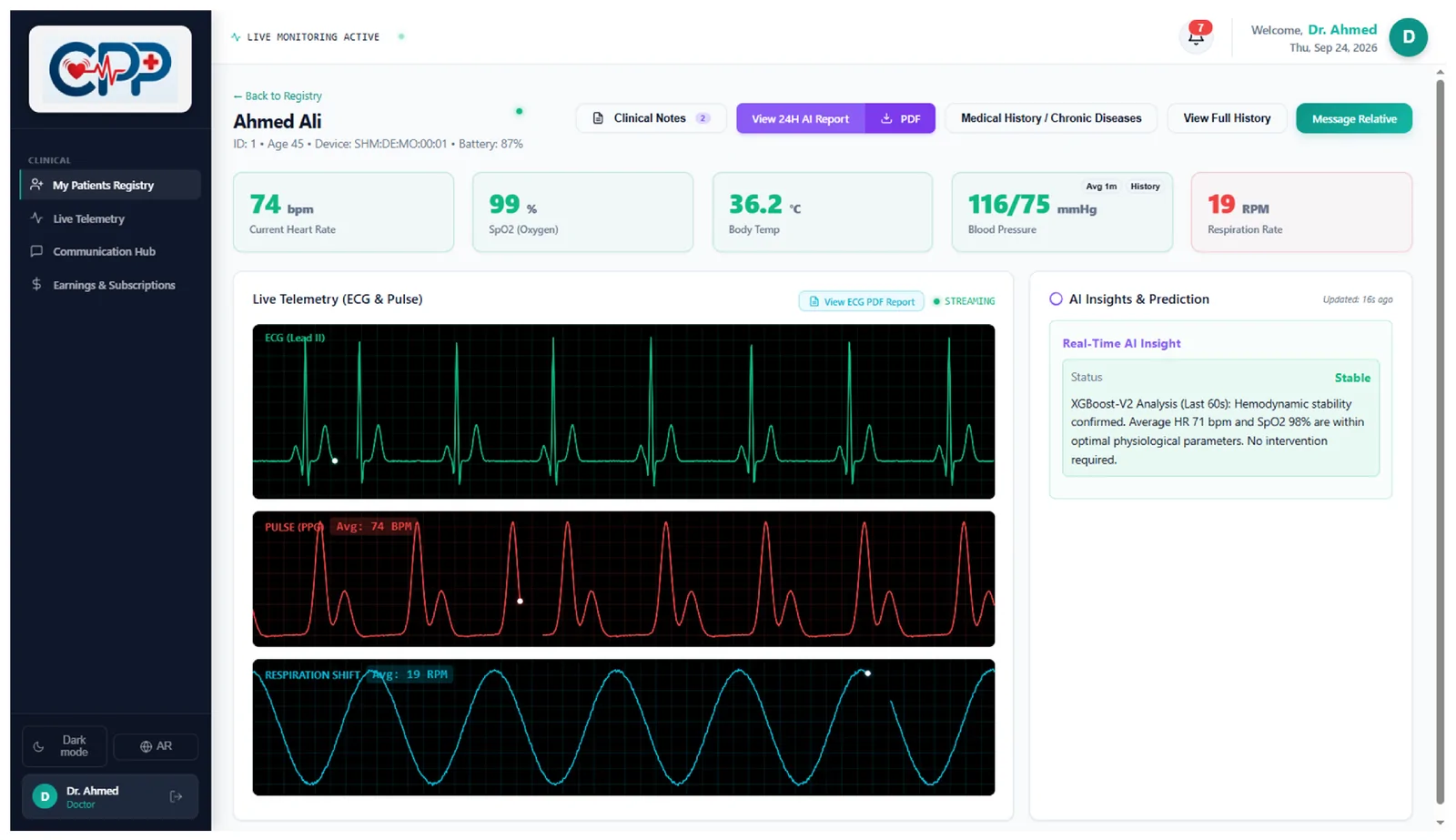
Clinician patient-detail dashboard showing synthetic vital-sign cards, waveform trends, and the platform’s decision-support panel.

**Figure 6:**
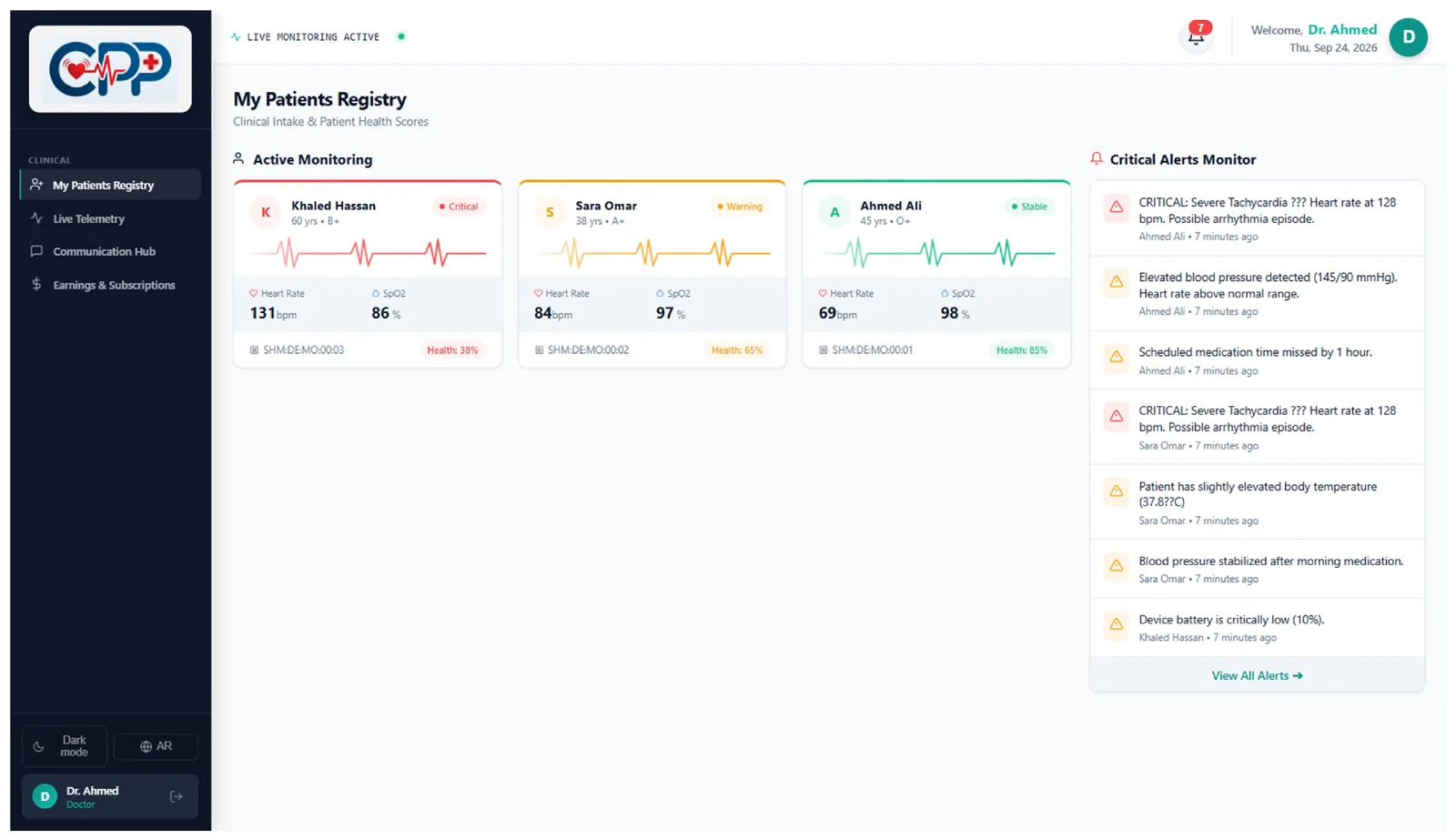
Doctor Dashboard, presenting synthetic demonstration records as risk-colored cards with live-status information and an at-a-glance critical-alert monitor.

**Figure 7:**
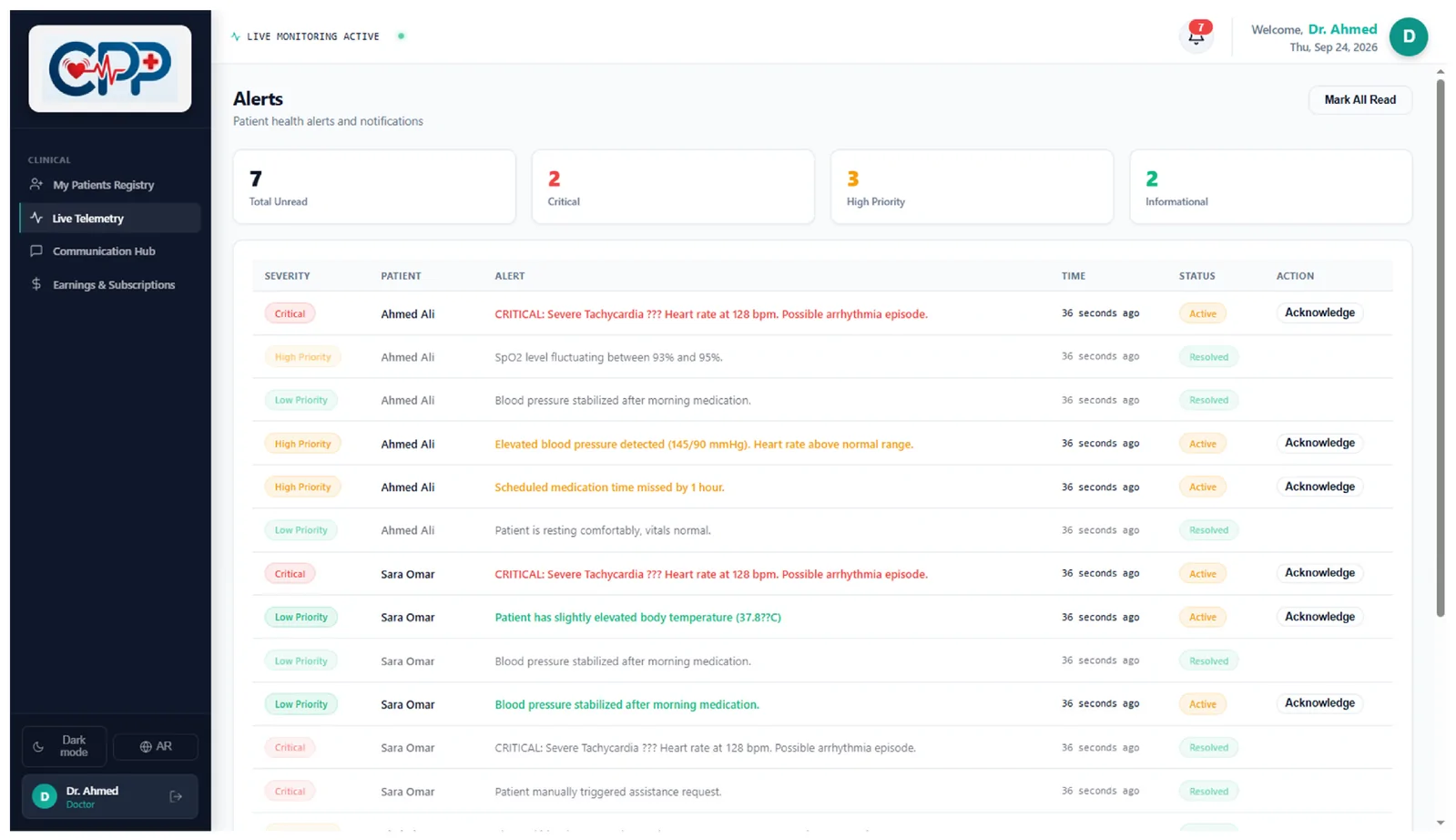
Alerts page, the clinician’s filterable inbox for synthetic alerts, with severity categories and acknowledgement actions.

**Table 1:** (**A**) Position of the present system relative to closely related architectures. (**B**) Scientific contribution of the previous system versus the present system.

**(A)**				
**System**	**Physiological Sensing**	**Remote Platform**	**Cuffless BP Timing**	**Automated Analytics**
Heaney et al. [7]	ECG + HR/SpO2 + temperature	Mobile/web + MATLAB	No	No
Georgieva-Tsaneva et al. [8]	ECG + PPG + temperature	IoT cardiac platform	No	HRV/arrhythmia analysis
Contardi et al. [9]	MAX30102 HR/SpO2	ESP32-native web server	No	No
Adochiei et al. [10]	ECG + PPG + temperature + SpO2	HL7 FHIR/MQTT/dashboards	No	No
Torad et al. [11]	ECG + SpO2 + temperature + air-quality sensing	Firebase + LabVIEW + Flutter	No	1D-CNN ECG classification
Present system	ECG + PPG/SpO2 + contact temperature	Laravel web platform	ECG-to-PPG PAT tracking/planned calibrated trend fields	Functional cloud triage (synthetic pipeline testing)
**(B)**				
**Comparison Dimension**	**Earlier Sensors Prototype [11]**		**Present System**	
Embedded architecture	Arduino acquisition plus Raspberry Pi edge processing		Single ESP32-S3 acquisition/network gateway with FreeRTOS dual-core temporal isolation	
Physiological timing feature	No cuffless blood-pressure timing feature		Shared-clock ECG-to-PPG PAT tracking; participant-specific pressure calibration is not validated in this manuscript	
Cloud/software architecture	Firebase + LabVIEW + Flutter; 1D-CNN ECG arrhythmia classification		Laravel browser platform + separately deployed FastAPI functional triage microservice	
Evidence/claim boundary	MIT-BIH-based classifier; source-reported sub-200 ms inference and 300–800 ms cloud latency		Bench-top engineering integration, synthetic triage-path testing, and qualitative endurance; no human-participant results are analyzed in this manuscript	

**Table 2:** Principal hardware channels and interfaces implemented in the prototype.

Component	Measured/Derived Quantity	Interface	ESP32-S3 Connection
AD8232	Single-lead ECG waveform	Analog + leads-off	GPIO 4 analog; GPIO 5 and 18 LO+/LO−
MAX30102	PPG, heart rate, SpO2	I2C	GPIO 8 SDA; GPIO 9 SCL
MAX30205	Contact skin/body-surface temperature	I2C	GPIO 8 SDA; GPIO 9 SCL
18650 subsystem	Battery supply/charge state	Analog power monitoring	ADC1-based battery sensing
RGB LEDs + buzzer	Local system/battery/alert status	Digital GPIO	Firmware-controlled

**Table 3:** Ten input features used by the cloud-triage classifier.

Feature	Type/Source	Role in Triage Vector
Age	Patient record	Demographic context
Gender	Patient record	Demographic context
Heart rate	MAX30102-derived	Current physiology
SpO2	MAX30102-derived	Current physiology
Systolic BP trend estimate	Planned PAT-derived trend field; deployed provenance not independently verified	Current/trend physiology
Diastolic BP trend estimate	Planned PAT-derived trend field; deployed provenance not independently verified	Current/trend physiology
Contact temperature	MAX30205	Current physiology
Diabetes status	Patient record	Comorbidity flag
Hypertension status	Patient record	Comorbidity flag
Smoker status	Patient record	Risk-factor flag

**Table 4:** Current validation status of physiological channels in the engineering proof of concept.

Channel	Sensor	Current Assessment	Current Manuscript Result	Interpretation
Heart rate	MAX30102	Reference wearable/monitor required for future validation	No paired reference comparison reported	Engineering sensing only; accuracy unestablished
SpO2	MAX30102	Reference oximetry device required for future validation	No paired reference comparison reported	Engineering sensing only; accuracy unestablished
ECG waveform	AD8232	Signal acquisition and leads-off behavior checked	No quantitative waveform agreement statistic	Engineering functional verification only
Contact temperature	MAX30205	Reference thermometer required for future validation	±0.1 °C manufacturer-rated sensor specification only	Not a measured system-level accuracy result

**Table 5:** Source-reported context relative to selected prior work. Protocols, subjects, reference instruments, endpoints, and test conditions differ; values are not a controlled head-to-head benchmark.

System	Published/Reported Comparison Figure
Heaney et al. [7]	99.56% accuracy versus the commercial reference device used in that study (as reported by the source)
Adochiei et al. [10]	Sub-second end-to-end data flow and zero packet loss across 10 acquisition sessions (as reported by the source)
Torad et al. [11]	<200 ms 1D-CNN inference and 300–800 ms round-trip cloud latency (as reported by the source)
Present proof of concept	Functional synthetic critical-input-to-dashboard presentation summarized as sub-second in development logs; no raw latency distribution or physiological accuracy result

## Data Availability

No human-participant dataset is analyzed or reported with this proof-of-concept manuscript. Non-sensitive engineering implementation records and project documentation may be made available by the corresponding author on reasonable request, subject to institutional, ethics, confidentiality, and intellectual-property requirements.

## Supplementary Materials

The following supporting information can be downloaded at: https://www.mdpi.com/article/10.3390/s26196047/s1, Figure S1: Patient Medical File page, allowing authorized clinicians to view and update demographic, chronic-condition, and medical-history fields used by the platform. Figure S2: Clinician Communication Hub, providing secure real-time messaging with patients’ relatives. Figure S3: Relative Communication Hub, providing the family-facing side of secure doctor-relative messaging. Figure S4: Administrator Executive Dashboard, summarizing platform-wide status, device-health information, audit activity, and infrastructure indicators. Figure S5: Doctors & Patients Lifecycle page, supporting doctor verification and patient-record/assignment oversight. Figure S6: Device Inventory Management page, used to register ESP32-S3 units and track assignment and telemetry status. Figure S7: My Subscription Plan page, the relative-facing interface for viewing and managing the hardware-rental subscription.
