# Supplementary material for: ESP32-S3-Based Multimodal Wearable for Remote Vital-Sign Acquisition with ECG-to-PPG Pulse-Arrival-Time Tracking and Functional Cloud Triage: An Engineering Proof of Concept
Source: Sensors (Basel). 2026 Sep 24;26(19):6047. doi: 10.3390/s26196047 (PMC13667402; doi:10.3390/s26196047)
Supplement: Supplementary file 1 [file sensors-26-06047-s001.zip › sensors-4558579-supplementary.pdf]

Supplementary Material: Interface Figures

Paper ID: sensors-4558579

The interface screens below were moved from the main manuscript to reduce screenshot-heavy presentation. All names, identifiers, physiological values, and alert labels shown are synthetic demonstration records used solely to document platform functionality; they are not human-participant data.

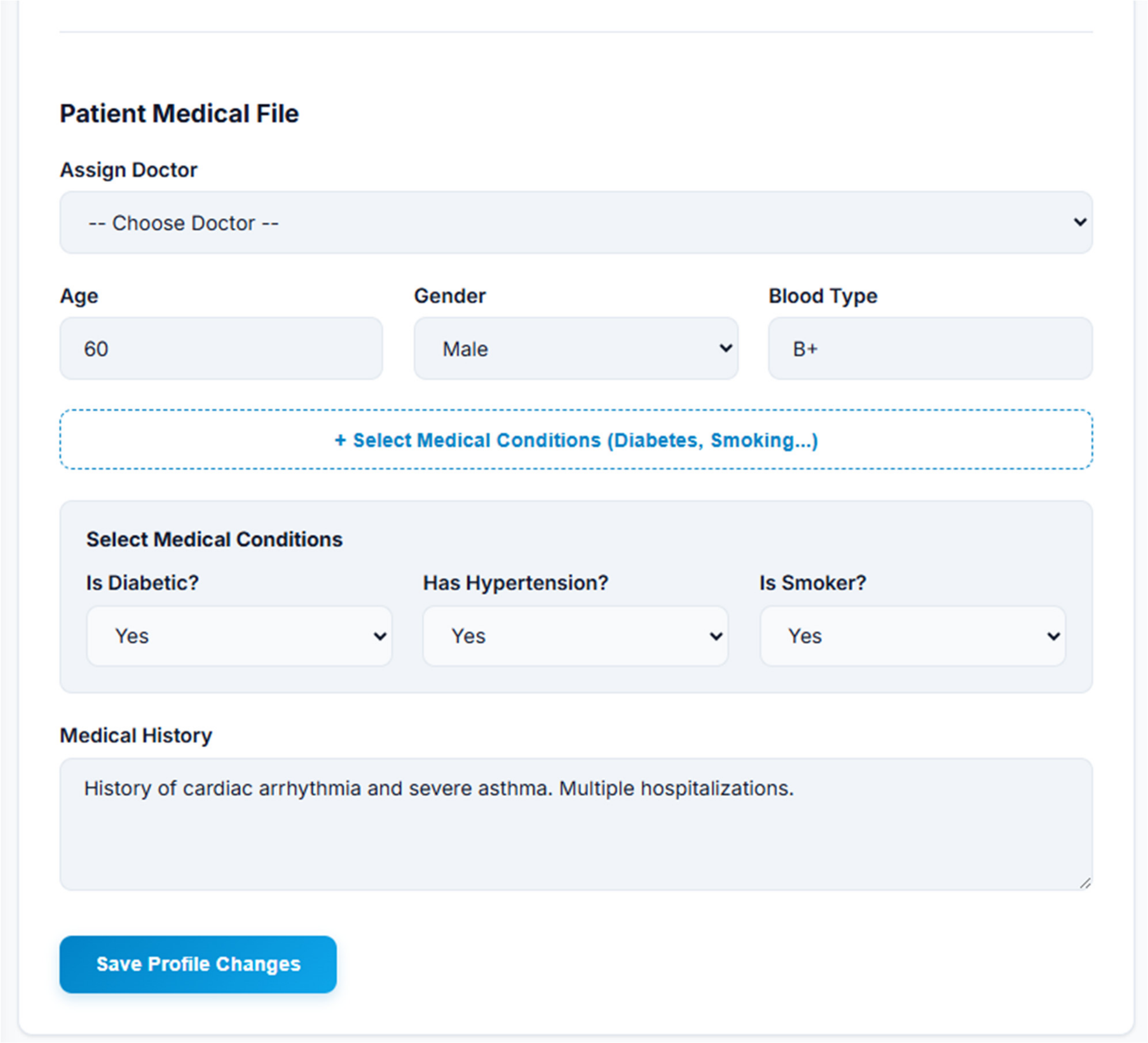

Figure S1. Patient Medical File page, allowing authorized clinicians to view and update demographic, chronic-condition, and medical-history fields used by the platform.

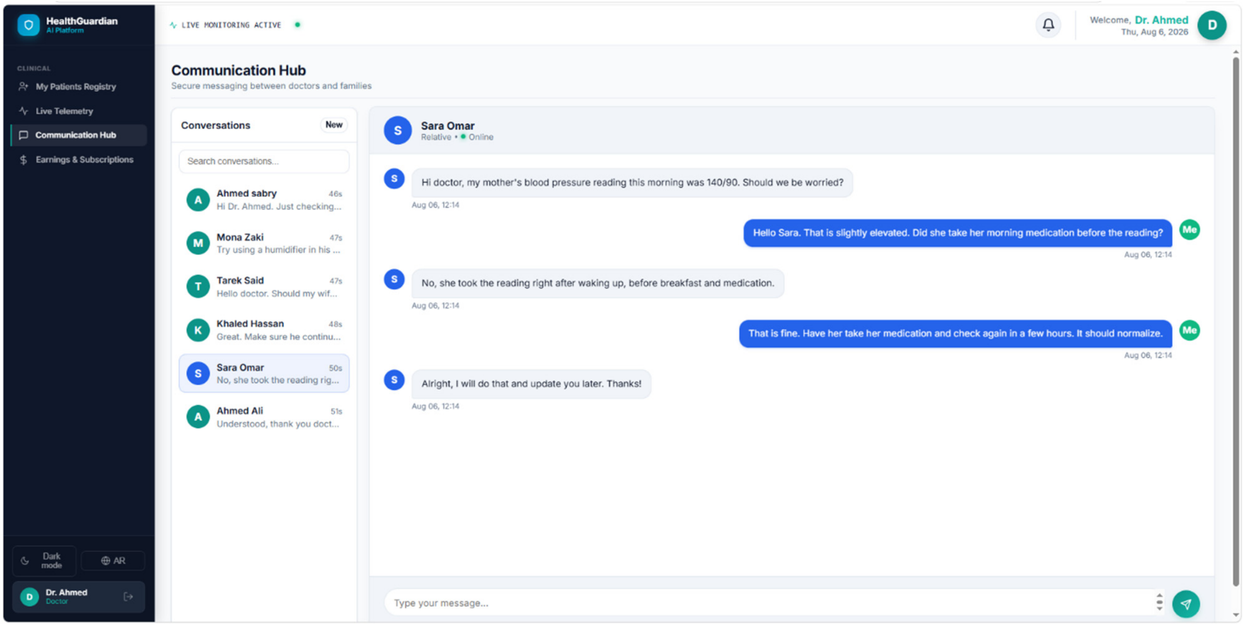

Figure S2. Clinician Communication Hub, providing secure real-time messaging with patients' relatives.

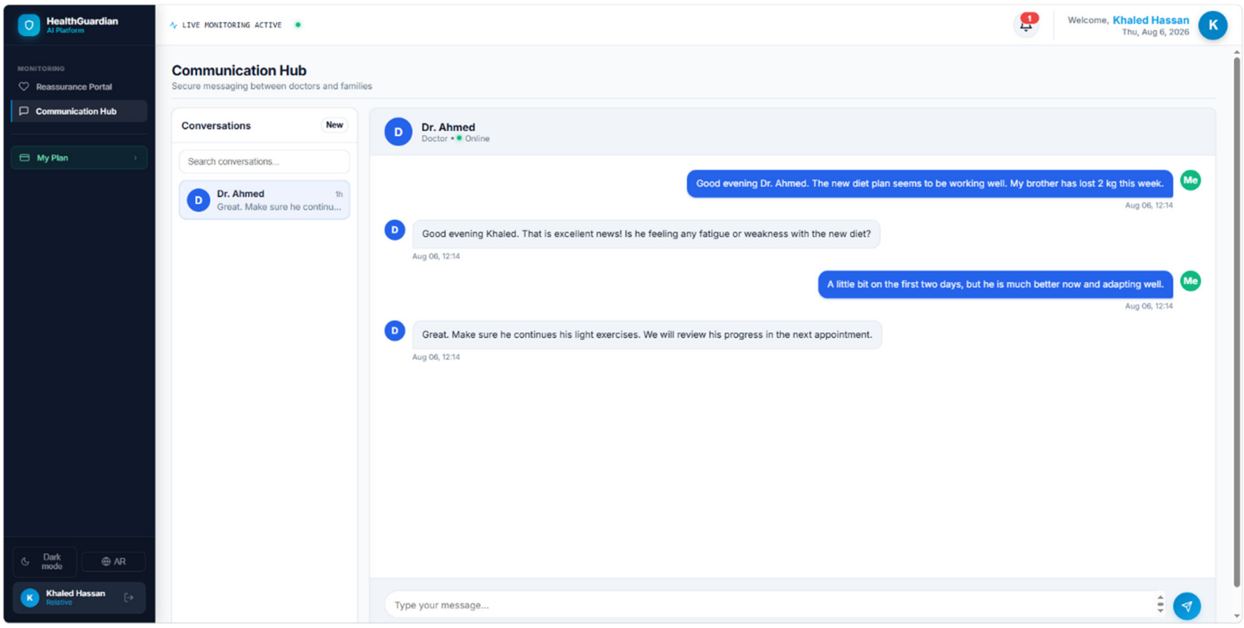

Figure S3. Relative Communication Hub, providing the family-facing side of secure doctor-relative messaging.

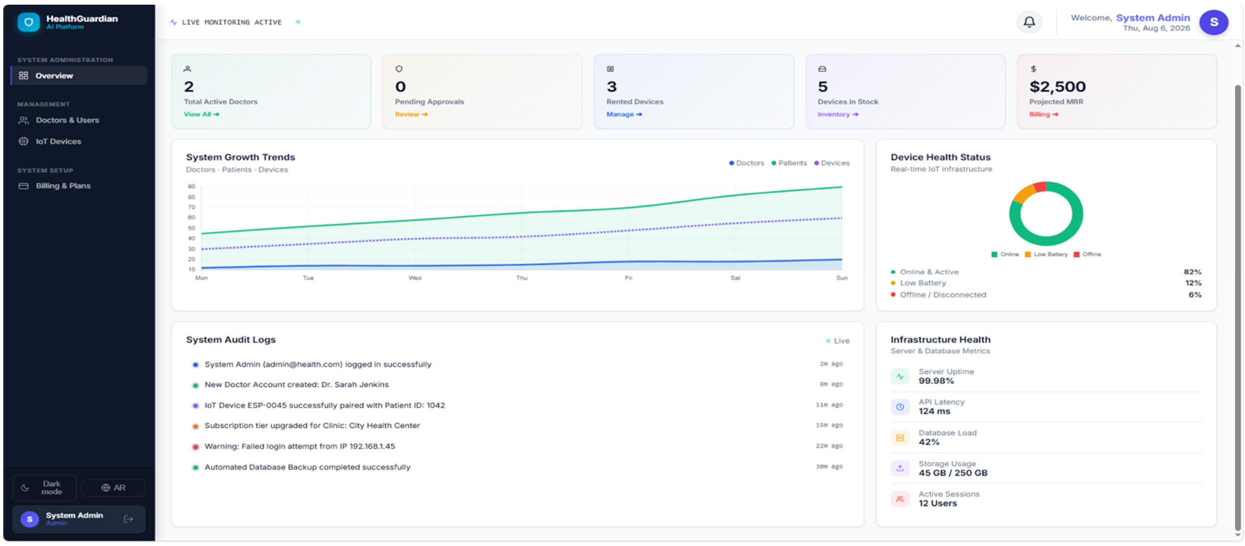

Figure S4. Administrator Executive Dashboard, summarizing platform-wide status, device-health information, audit activity, and infrastructure indicators.

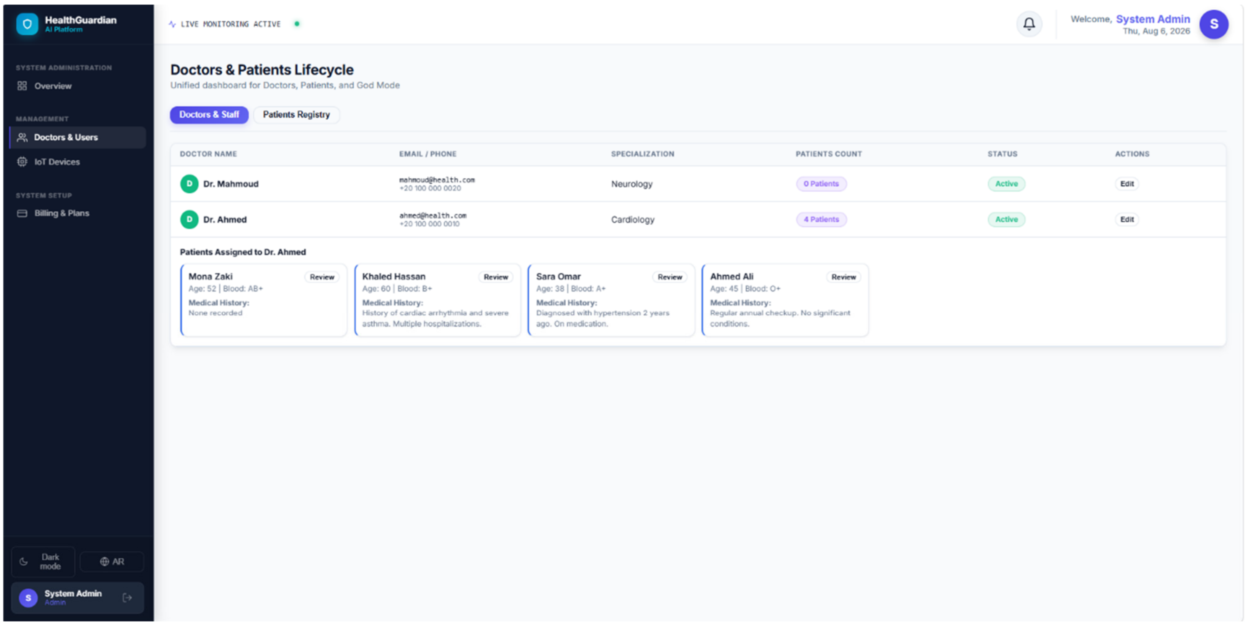

Figure S5. Doctors & Patients Lifecycle page, supporting doctor verification and patient-record/assignment oversight.

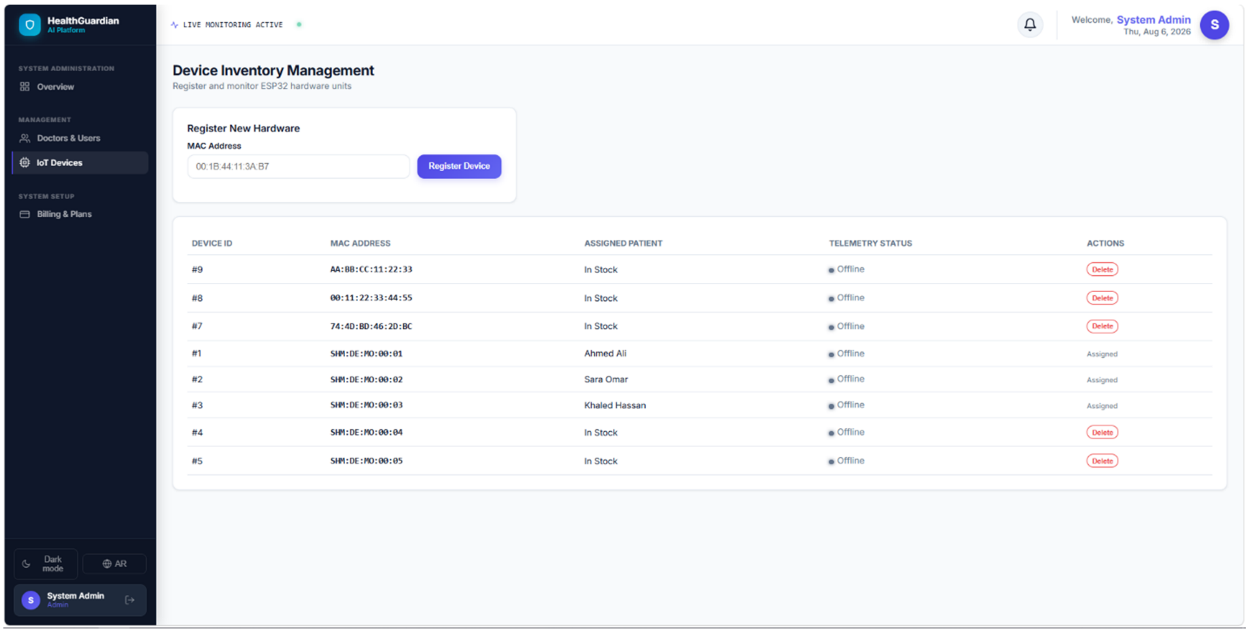

Figure S6. Device Inventory Management page, used to register ESP32-S3 units and track assignment and telemetry status.

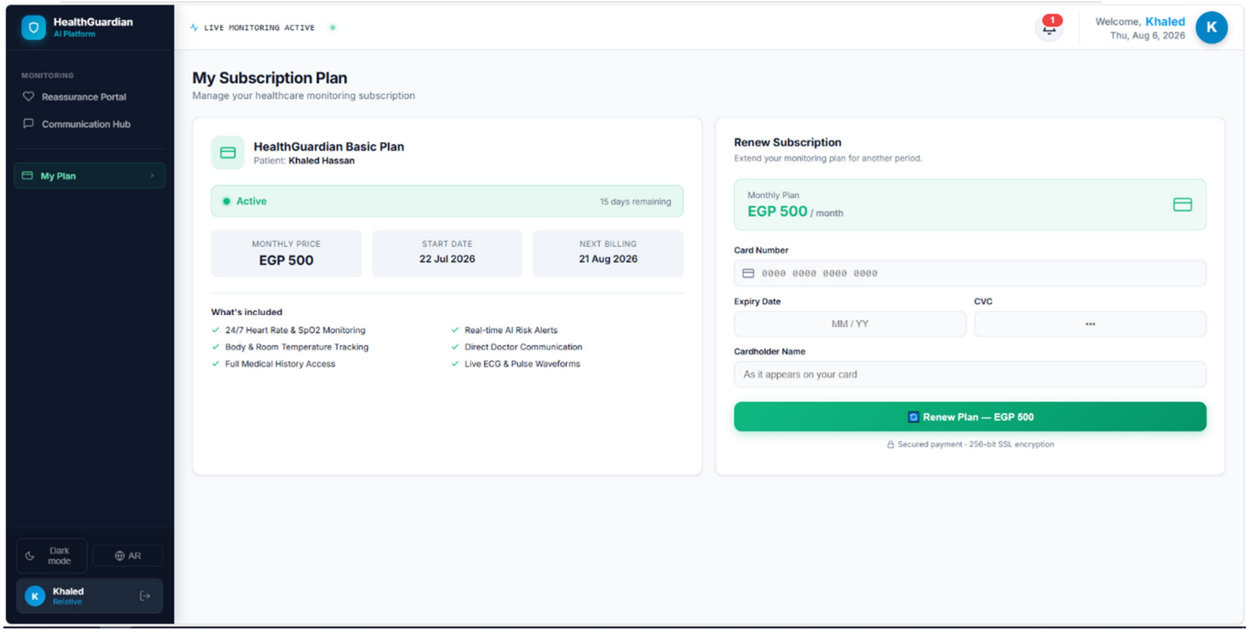

Figure S7. My Subscription Plan page, the relative-facing interface for viewing and managing the hardware-rental subscription.
